# Special Issue “Decision Models in Green Growth and Sustainable Development”

**DOI:** 10.3390/ijerph15061093

**Published:** 2018-05-28

**Authors:** Ning Zhang, Zaiwu Gong, Kedong Yin, Yuhong Wang

**Affiliations:** 1 School of Management Science and Engineering, Nanjing University of Information Science and Technology, Nanjing 210044, China; lyguangzhiying@163.com (N.Z.); zwgong26@163.com (Z.G.); 2 Collaborative Innovation Center on Forecast and Evaluation of Meteorological Disasters, Nanjing 210044, China; 3 School of Economics, Ocean University of China, Qingdao 266100, Shandong, China; 4 Institute of Marine Development, Ocean University of China, Qingdao 266100, Shandong, China; 5 School of Business, Jiangnan University, Wuxi 214122, Jiangsu, China; yuhongwang@jiangnan.edu.cn

**Keywords:** sustainable development, decision models, disaster prevention and mitigation, sustainable operations, supply chain management

In the report issued by the World Commission on Environment and Development (WCED) in 1987, “Our Common Future”, sustainable development was clearly defined as “a development that meets the needs of the current generation without compromising the ability of future generations to meet their needs” [1]. Sustainable development is mainly concerned with human, economy, society, resources and environment, and different disciplines furnish different perspectives and angles. From an economics perspective, this discipline studies issues ranging from regional development, productivity distribution, economic structure optimization, to the balance of supply and demand. The aim is to establish proper development models for maximizing the efficiency of resource utilization under environmental and societal constraints [2]. From an ecological perspective, sustainable development is a development that does not exceed the regeneration capacity of environmental systems, and the research focuses on finding the best ecosystem to support the integrity of ecology and the realization of human aspirations, which is the sustainability of the human environment [3]. On the other hand, in sociological research, the focus is the equity and efficiency of sustainable development, emphasizing sustainable development of the society through proper development strategies [3].

In the contemporary society, the 2017 G20 summit identified a main theme of “Building economic resilience for sustainability and accountability”, and declared that “strong, sustainable, balanced, and inclusive growth” is a global priority. Especially, at the 2016 G20 summit in Hangzhou, world leaders reached a consensus that sustainable development is one of the three main goals. Sustainable development has become one of the most urgent and serious challenges in the world. An ever increasing trend is to introduce technology for green growth and promoting sustainable development. The introduction of quantitative research methods in economics and the innovative use of “cost-benefit” qualitative research methods in traditional economics make it possible for such sustainable development ideas as circular economics. Overexploitation of traditional energy sources such as coal and oil has led to significant environmental pollution and ecological degradation, which creates an urgent need to develop new green energy sources, such as wind, solar, hydro, and nuclear power to alleviate ecological pressure and reduce carbon and environmental footprints, thereby realizing environmental and ecological sustainability. On the other hand, sustainable development and employment, sustainable development evaluation, green supply chain management and other relevant research topics have provided novel angles for examining sustainability of social systems.

As stated in the declaration of the Hamburg summit in Germany, “Shaping an interconnected world”, achieving the goal of sustainable development requires full cooperation of human beings from all over the world. It is inevitable that multiple stakeholders with different interests and perspectives must be involved in coming up with proper sustainable development strategies and their implementation. To this end, different decision models such as quantitative and qualitative economic analysis, environmental impact assessment, and sustainable development assessment can provide novel angles and innovative approaches to promote green growth and sustainable development.

## 1. Motivations for Organizing This Special Issue

This Special Issue was conceived to address various decision modeling issues in green development and sustainable development. More specifically, the following considerations motivate us to propose this Special Issue.

(1) Population, resources, environment and development, or “PRED” for short, are inter-related and can be mutual restraint. A viable way of achieving sustainable development is to maintain a balance among the “PRED”. Rapid population growth, shortage of natural resources, and deterioration of ecological environment have become critical obstacles of sustainable development. A conventional wisdom is that social development cannot exceed the carrying capacity of resources and environment, which embodies the sustainability principle. In the meanwhile, sustainable development calls for equity in opportunities and benefits: current development in a region cannot be accomplished at the expense of the development of other regions or future generations, reflecting the fairness principle. Finally, sustainable development is recognized as a global problem beyond national boundaries and cultural and historical barriers, and requires the cooperation of the whole world, embodying the commonality principle. Under these three basic principles of sustainable development, sensible solutions to the “PRED” problem require a cross-disciplinary perspective and take an integrative view from such disciplines as ecology, economics, and sociology.
(2) Sustainable supply chain management is a subset of sustainable development, focusing on sustainable operations of supply chain systems [4]. Sustainable supply chain management calls for systematic integration and coordination of material, information, and financial flows for maximizing the profitability of the entire supply chain, as well as optimizing the environmental and social benefits; Generally speaking, sustainable operations of supply chains requires that: (1) the long-term strategic goals of the member enterprises must be compatible in economic, environmental and social dimensions; (2) The whole supply chain system has to be balanced to ensure sustainable operations and profitability. To promote sustainable supply chain management, academics have been carrying out research along several lines such as green supply chain operations, core competence and implementation strategies.
(3) Sustainable development research often establishes effective mathematical models and analytical methods, including quantitative and qualitative economic models, group decision making (GDM), and conflict analysis. Many critical sustainable development issues such as global climate change, nuclear power plant construction and maintenance, among others require the public to launch extensive and adequate discussions so that meaningful input can be provided to the policy makers. Different stakeholders often go through multiple rounds of negotiation to reach a compromise. To reach a consensus, some even all of the stakeholders may have to modify their positions. To properly characterize this GDM process and obtain optimal ranking of available alternatives, different decision tools such as goal programming, analytic hierarchy process (AHP), and information aggregation are proved indispensable.

## 2. About the Papers of This Special Issue

In this Special Issue, 54 papers have been accepted as full papers. A wide range of sustainable development issues have been addressed such as:

(1) To satisfy human needs for sustainable development, many constraints concerning natural resources and environment must be accounted for. According to the data from the main food production provinces, Zhang et al. [5] adopted a stochastic frontier analysis methodology to study sustainable development of food quality in China. Taking regulation as a mean to reducing negative externality and enhancing positive externality, Wang et al. [6] proposed an improved ordering preference method with entropy weight and Mahalanobis distance (E-M-TOPSIS) to conduct a performance evaluation of China’s energy regulation. Using the reservoir resettlement project of the Wujiang River cascade hydropower station as a case study, Huang et al. [7] established a set of evaluation index system for assessing sustainable development of resettlers based on a back propagation neural network. Wu et al. [8] forecasted water demand in Chongqing by employing a grey forecasting model and put forward useful suggestions for sustainable development of urban water consumption. Bai et al. [9] evaluated four architectural structures in rural China by using an AHP-Grey correlation analysis, thereby selecting the most appropriate structure insulated panels (SIPs) for promoting sustainable development. Li et al. [10] constructed a compensation standard model for regional forest ecosystems, and applied it to the forest ecosystem in Yanqing District, Beijing, China.
(2) Environmental pollution has seriously affected industrial and agricultural production and human life, and environmental regulation provides policy guidelines for environmental sustainable development. According to Chinese manufacturing industries data, Hu et al. [11] explored the spillover effect of environmental regulation and foreign direct investment (FDI) on green technology progress. Gao and Zheng [12] adopted a three-stage Stackelberg game model to examine the interaction between a profit-maximizing firm (Stackelberg leader) with emission-dependent demand and an environmental regulatory agency (Stackelberg follower). Jiang et al. [13] developed a denitrification decomposition (DNDC) model to simulate and evaluate the agricultural carbon sequestration and emission reduction process in agriculture in China. Li et al. [14] predicted annual wastewater discharge from four primary pollution sources in the Three Gorges Reservoir area by an optimized GM(1,1) model, and put forward several countermeasures for water pollution control and sustainable development in the Three Gorges Reservoir area. Along a similar line, Li et al. [15] explored the optimal fractional order grey model to forecast the amount of sewage discharge in the Yangtze River Basin, with a purpose of promoting the sustainable development of the Yangtze River. Zhou et al. [16] employed a hybrid approach combining with data envelopment analysis to determine key pollution factors causing lung cancer and identify whether rural or urban residents are more vulnerable to air pollution by using the data from Nanchang, China. In view of the Environment Kuznets Curve (EKC) and data from China in 1995–2012, Jin et al. [17] studied the relationship between technological progress and carbon emission. Based on the data of carbon dioxide emission in Chongqing, Yang et al. [18] analyzed and forecasted other fossil energy consumption and corresponding CO${}_{2}$ emission in 2020.
(3) Economic, ecological environment and social subsystems are interrelated and mutually restrictive, and holistic coordinated development is in accord with the principle of sustainability and fairness. Wang et al. [19] adopted panel smooth transition regression (PSTR) models to investigate the non-linear relationship between provincial economic growth and carbon emissions in China. Yuan et al. [20] evaluated the interactions among energy consumption, CO${}_{2}$ emission and growth patterns of the Chinese provincial transportation sector, thereby offering policy implications on provincial transportation sustainable development. Lu [21] investigated the co-movement and causality between greenhouse gas emissions, energy consumption and economic growth based on the data from 16 Asian countries between 1990–2012. By constructing a financial development index system, Xing et al. [22] studied the long-term relationship between financial development and carbon emissions based on a STIRPAT (Stochastic impacts by regression on population, affluence, and technology) model. By analyzing regional system structure and functions, Liang et al. [23] constructed an evaluation index system, consisting of economic, ecological, environmental and social factors, to assess the capacity of sustainable development in Sichuan province. Based on a generalized method of moments (GMM), Cao et al. [24] investigated the impact of environmental regulation on employment in five typical resource-based provinces in China from 2000 to 2015. Using data from the textile industry, Xu [25] proposed some recommendations for sustainable development of China’s textile exports. Based on a case study with a stochastically improving energy saving technology and a stochastically rising energy price, Chen et al. [26] explored the impact of a sustainable development technology on different macroeconomic variables of a small economy. Xiao et al. [27] established a two-stage dynamic game model to examine the impact of greenhouse gas emission regulations and financial development on the supply chain operations. Based on the nonlinear least square (NLS) method, Pei et al. [28] constructed a novel grey multivariable model, and investigated the relationship between pollutant discharge and economic growth.
(4) Sustainable supply chain management is an important research topic in supply chain management. Based on the concept of cooperative advertising investment, Feng and Liu [29] constructed a manufacturer-led supply chain structure and analyzed how online word-of-mouth (OWOM) affect supply chain performance. By using a spatial Durbin model, Xu and Wang [30] studied the direct impact of logistics development on economic growth and the spatial spillover effect. Based on a two-echelon supply chain with one manufacturer and one retailer, Liu et al. [31] investigated joint decision-making and coordination of the supply chain system under carbon tax regulation and fairness concerns. Based on social welfare maximization, Chen and Su [32] established two game theoretic models for photo voltaic supply chains with and without public subsidy, where the two supply chains may take a completely centralized, completely decentralized, or hybrid strategy. Li et al. [33] compared and analyzed the optimal decisions in a low-carbon closed-loop supply chain under vertical and horizontal cooperation modes, and proposed schemes for carbon emission reduction and profit maximization. Zhu et al. [34] constructed a dual-channel closed-loop supply chain model composed of manufacturers, retailers, and network recycling platforms, and investigated the influence of consumer bargaining behavior on the recycling process by employing the Stackelberg game theory. Based on the two-factor theory, Wang et al. [35] sampled middle-level and low-level managers of new energy enterprises, and studied the effect of internal and external incentive factors on management performance from an empirical perspective. To address optimization of a fresh food logistics distribution system, Wang et al. [36] developed a green and low-carbon location-routing problem (LRP) model in cold chain logistics. A hybrid genetic algorithm with heuristic rules is devised to solve this cost minimization model. Taking a particular emergency logistics network as an example, Wang et al. [37] simulated and analyzed the network reliability under two different attack modes. This research furnished a new tool for reliability research of complex emergency logistics networks.
(5) Disasters affect sustainable development of society, and research on disaster prevention and mitigation (including disaster risk, emergency response and post-disaster management) contributes to post-disaster recovery and sustainable development of human society. Towe et al. [38] made a detailed classification of NGOs and their services to achieve the goal of optimizing disaster regulation. Yin et al. [39] established a grey relational model based on dispersion of panel data, and studied the loss of rainstorm disasters in China. From a statistical distribution perspective, Wang and Gong [40] investigated the optimal ranking of hesitant fuzzy linguistic preference relations based on chance constrained programming with an application to meteorological disaster risk assessment. Considering the evaluation for meteorological disasters, Yu et al. [41] constructed a multiple-criteria evaluation model based on novel evaluation algorithm. Jin et al. [42] applied econometric model groups in assessment of Chinese storm surge disaster damage.
(6) From a modeling perspective, sustainable development needs to establish effective mathematical models and analytical methods, such as multi-attribute decision making, conflict analysis, and GDM. Sie et al. [43] constructed a group model to promote consensus, and applied in acquiring opinions consensus for multi-use deep water offshore platforms. To solve the problem of multi-attribute GDM, Yin et al. [44] proposed two aggregation operators based on the trapezoidal fuzzy two-dimensional linguistic weighted partitioned Bonferroni mean (TF2DLWPBM). In another article, Yin et al. [45] put forward an improved grey correlation multi-attribute GDM method to handle interval grey trapezoid fuzzy linguistic variables and unknown weights. Liu et al. [46] employed a multi-attribute decision making method based on intuitionistic linguistic information to investigate the low carbon supplier selection problem. Based on option prioritization, Yin et al. [47] established an improved graph model for conflict resolution to examine the Changzhou brownfield conflict. Qu and Zhou [48] studied the effect of uncertain demand on low-carbon products by using a newsvendor model. Taking into account of the sensitivity of various microorganisms to ozone, Brodowska et al. [49] constructed a microbial model to preserve different strains of food. Considering the hesitancy of decision-making problems and the priority of evaluation criteria, Qi et al. [50] adopted a fuzzy GDM method to deal with the evaluation of complex emergency response solutions. Lin and Wang [51] constructed a linguistic multi-attribute GDM linear model based on risk preference with an application to the selection of low-carbon tourist destinations. Based on multiplicative and additive consistency, Gong and Wang [52] studied the measurement of individual consistency and group consensus of fuzzy preference relations. Sun et al. [53] developed a new decision-making method based on grey multi-source heterogeneous data and applied it to the green supplier selection problem. By introducing the kernel and grey scale, Li et al. [54] explored a grey linguistic hesitant fuzzy GDM method and employed it to evaluate the sustainable development ability of industry chains. By incorporating strengths of preference into a graph model, Yu and Pei [55] carried out a systematic investigation of a brownfield redevelopment conflict occurred in Changzhou, China. Using decision-making method, Chen et al. [56] investigated that cap-and-trade and take-back regulations have a positive impact on environment. Liao et al. [57] combined hesitant fuzzy linguistic preference relations with utility functions, and put forward effective GDM models for emergency management. For the dynamic uncertain system of urban water demand, Li et al. [58] explored a robust economic control decision method with a variety of variables.
